# Cathepsins effect on diffuse large B cell lymphoma risk: A Mendelian randomization study

**DOI:** 10.1097/MD.0000000000046485

**Published:** 2026-05-12

**Authors:** Chuanyang Lu, Qiuni Chen, Yuye Shi, Yuan Deng, Chunling Wang, Liang Yu

**Affiliations:** a Department of Hematology, The Affiliated Huaian No.1 People’s Hospital of Nanjing Medical University, Huaian, Jiangsu Province, China; b Key Laboratory of Autoimmune Diseases of Huaian City, Huaian, Jiangsu Province, China; c Key Laboratory of Hematology of Nanjing Medical University, Nanjing, Jiangsu Province, China.

**Keywords:** cathepsin, diffuse large B cell lymphoma, instrumental variants, Mendelian randomization, single nucleotide polymorphisms

## Abstract

Recently, the relationship between cathepsins and a variety of diseases has gained increasing attention, especially various solid tumors and hematologic malignancies. However, there is no consensus on the causality between cathepsins and malignant lymphomas. The cathepsin genome-wide association studies (GWAS) dataset for this study was derived from THE INTERVAL STUDY and was freely downloaded via the OPENGWAS website. GWAS summary statistics for lymphomas were obtained from the FinnGen database. Single nucleotide polymorphisms satisfying the genome-wide association significance (*P* < 5 × 10^−6^) and linkage disequilibrium parameters (*r*^2^ = 0.001 and clumping distance = 10,000 kb) were screened for the subsequent 2-sample Mendelian randomization (MR) analysis. Sensitivity analyses were utilized to assess the stability and robustness of the MR conclusions. Genetically predicted Cathepsin S (CTSS) level was positively associated with the increased risk of diffuse large B cell lymphoma (DLBCL). Patients with elevated levels of CTSS are more susceptible to DLBCL, which corresponds to a 20.6% increased hazard (odds ratio inverse variance weighted [OR_IVW_] = 1.206, 95% confidence interval [CI] 1.054–1.380, *P* = .006). The causal role of the remaining cathepsins in DLBCL and other types of lymphoma was not established. No reverse causality between DLBCL and CTSS was observed after the backward MR analysis (OR_IVW_ = 1.076, 95% CI 0.984–1.177, *P* = .109). The multivariate MR results supported that CTSS was causally responsible for DLBCL (OR_IVW_ = 1.204, 95% CI 1.042–1.392, *P* = .012, OR_MR-Egger_ = 1.200, 95% CI 1.038–1.387, *P* = .014). This study demonstrated a causal effect of CTSS on DLBCL based on the MR algorithm. Follow-up studies are needed to elucidate the underlying mechanisms between them and explore promising new therapeutic options for DLBCL patients.

## 1. Introduction

Malignant lymphomas are a heterogeneous group of hematologic malignancies originating in the lymph nodes and lymphoid tissues. Lymphomas are classified into 2 main groups including Hodgkin lymphoma (HL) and non-Hodgkin lymphoma (NHL) based on their histopathological features. Of these, NHL is the most common lymphoma with morbidity consistently 3 to 5 times higher than HL,^[1]^ and 2020 global cancer statistics revealed that NHL has one of the highest incidence and mortality rates. The number of new NHL cases was 544,352 and the number of deaths was 259,793 in 2020.^[2]^ The incidence of NHL was the 8th and 10th highest in men and women, respectively.^[3]^ NHL is also the second most common cause of death from hematologic malignancies, after leukemia. At the same time, about 30% to 40% of patients have to face the challenge of ineffectiveness to existing lymphoma treatment modalities or relapse after treatments.^[4]^ Thus, in summary, malignant lymphoma remains a significant global health burden that deserves greater attention for the researchers and internists worldwide.

Cathepsins are a collective term for a class of protein hydrolases, belonging to the papain superfamily of cysteine proteases, which are relatively conserved during biological evolution.^[5]^ They maintain optimal activity under slightly acidic pH conditions within the lysosome. Physiologically, their activation and catalytic activity are regulated by a sophisticated modulatory network that participates in tissue- and cell-specific biological processes including protein and lipid metabolism, antigen processing and presentation, autophagy, and lysosome-mediated cell death. One of the most prominent functions is the involvement in the regulation of the immune system.^[6]^ When any part of the regulatory network is deregulated, it will lead to overactivation, aberrant expression, or mislocalization and be involved in the pathogenesis of a wide range of diseases. Previous research has demonstrated that cathepsins are potential prognostic markers and therapeutic targets for a variety of diseases under pathological conditions.^[7,8]^ In recent years, the role of cathepsins in cancer development, progression, and dissemination has been increasingly recognized.^[9]^ It has been found that cathepsins can be used as prognostic markers in cancer progression and interact with the tumor microenvironment making them one of the targets for antitumor therapy resistance.^[10]^

Mendelian randomization (MR) analysis is a method of causal inference that has the advantage of eliminating the interference of confounding factors and reverse causal associations by contrast to traditional observational studies. Broadly speaking, MR studies center on inferring causal associations between the exposure and target outcome through genetic tools, usually single nucleotide polymorphisms (SNPs), that are strongly associated with the studied exposure variable.^[11]^ With the development of genome-wide association studies (GWAS), a number of susceptibility loci for diffuse large B cell lymphoma (DLBCL) have been reported to date.^[12,13]^ However, only few reports of correlation between cathepsins and hematologic malignancies have been reported^[10,14]^ and the underlying mechanism of the causality has not been fully elucidated. Therefore, this study aims to investigate the causal role of cathepsins in DLBCL using an MR analysis.

## 2. Materials and methods

### 2.1. Datasets for cathepsin and malignant lymphoma GWAS

The summary genetic dataset for the cathepsin GWAS was derived from THE INTERVAL STUDY, conducted by Sun et al.^[15]^ The cathepsin GWAS included 3301 individuals of European ancestry and comprised 10,534,735 genetic variables. A total of 9 cathepsins were enlisted in this MR study, namely Cathepsin B, E, F, G, H, O, S, L2, and Z. The above genetic statistics are publicly available on the OPENGWAS website (https://gwas.mrcieu.ac.uk/). The detailed descriptions of the malignant lymphoma GWAS were obtained from the FinnGen Consortium, which is obtainable through the FinnGen website (https://www.finngen.fi/).^[16]^ The outcome variable in this study was malignant lymphoma, including HL and NHL. NHL presents certain heterogeneity from HL in terms of histologic features, biological characteristics, immunophenotypes, clinical manifestations, therapeutic efficacy, and prognosis.^[17]^ Not only that, but this difference persists across different types of NHL and even within the same type of NHL such as DLBCL. Considering a large number of NHL classifications, the 4 most common subtypes including DLBCL, follicular lymphoma, marginal zone lymphoma, and mantel cell lymphoma were included in this study.^[18]^ The above-mentioned GWAS have already been approved by their respective ethical committees. Therefore, no additional ethical approvals were required for this study.

### 2.2. Genetic instrumental variants for cathepsin

**Figure 1.**
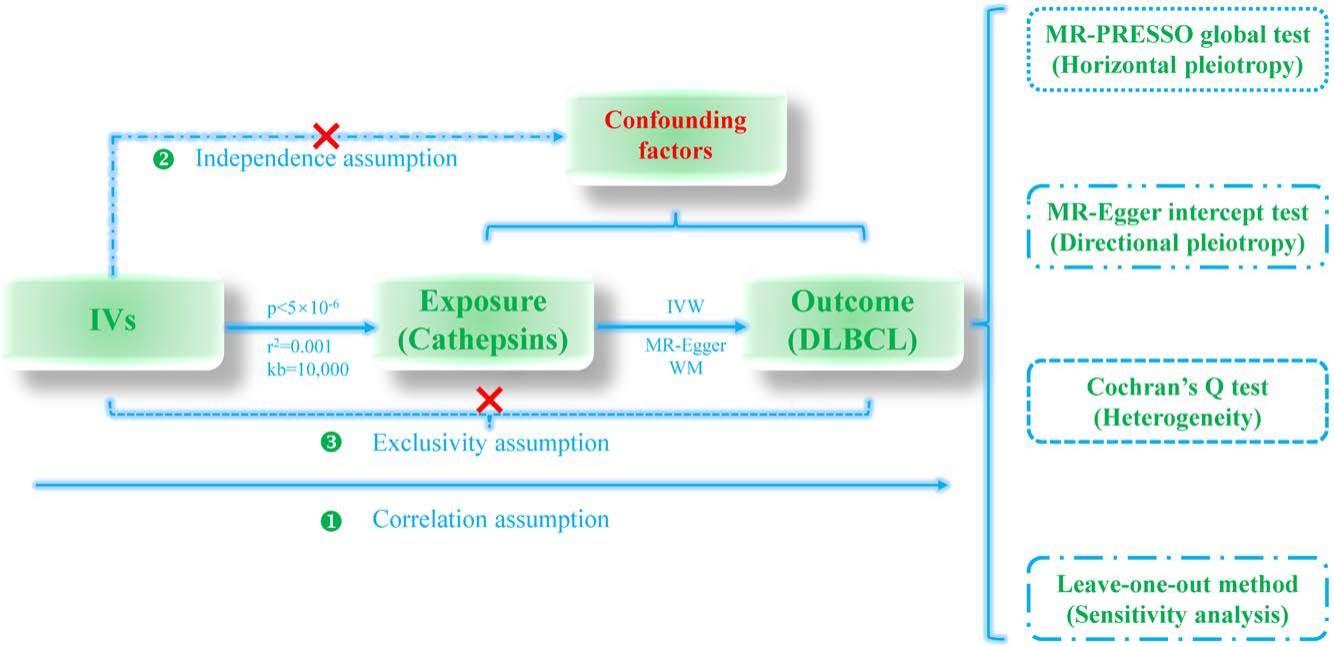
The schematic diagram of the flow of this study. Correlation assumption: genetic instrumental variants (IVs) are strongly associated with cathepsin; independence assumption: SNPs are independent of confounding factors; exclusivity assumption: there is one and only one pathway by which IVs can have an effect on lymphoma. The Cochran Q test, the MR-PRESSO global test, the MR-Egger intercept test, and the leave-one-out method were employed for sensitivity analyses. MR-PRESSO = MR Pleiotropy RESidual Sum and Outlier, SNPs = Single nucleotide polymorphisms.

The MR study was premised on 3 core assumptions, which are the correlation assumption: genetic instrumental variants (IVs) must be strongly associated with cathepsin; the independence assumption: SNPs are independent of confounding factors affecting cathepsin and malignant lymphoma; and the exclusivity assumption: there is one and only one pathway by which genetic variables can have an effect on lymphoma. Figure 1 visualized the aforementioned 3 central hypotheses. To ensure the correctness and robustness of causal inference, first, SNPs satisfying the genome-wide association significant level (*P* < 5 × 10^−6^) and the linkage disequilibrium parameter (*r*^2^ = 0.001, clumping window size = 10,000 kb) were selected from the cathepsin GWAS. Next, the MR Pleiotropy RESidual Sum and Outlier (MR-PRESSO) test was utilized to identify outlier SNPs.^[19]^ Both outliers and palindromic SNPs were filtered out. Finally, the F statistics of each genetic IV were calculated by the following formula: $F=\frac{N-k-1}{k}\times \frac{R^{2}}{1-R^{2\;}}$, where N represents the sample size in the cathepsin GWAS, *k* represents the number of IVs, and *R*^2^ represents the degree to which genetic variants explain exposure and was calculated as follows:$R^{2}=\frac{2\;\times \;\beta^{2}\times EAF\times \left(1-EAF\right)}{2\;\times \;\beta^{2}\times EAF\times \left(1-EAF\right)+2\times SE^{2}\times N\times EAF\times \left(1-EAF\right)}$).^[20,21]^ SNPs with F statistics >10 were considered to have no significant weak variable bias. The remaining IVs that fulfill the basic criteria were prepared for subsequent MR analyses.

### 2.3. The forward MR analysis

The workflow chart of this study was described in Figure 1. The statistical analyses of causal inference between cathepsin and malignant lymphoma were mainly performed using 3 models, respectively, MR-Egger,^[22]^ inverse variance weighted^[23,24]^ (IVW), and weighted median^[25]^ approaches. The odds ratio (OR) served as the overall effect value assessing the causality between cathepsin and malignant lymphoma. In case of inconsistent results among the 3 methods, the IVW model is preferred as a conclusion for causal inference. The 95% confidence interval (CI) for the OR excluded 1 and the corresponding *P*-value < .05 was considered statistically significant. At this point, OR > 1 indicates that cathepsin is a potential risk factor for malignant lymphoma, implying that individuals with elevated cathepsin levels have increased susceptibility to lymphoma. The MR-Robust Adjusted Profile Score method was conducted owing to its robustness to the violation of key assumptions underlying the MR study, such as pleiotropy and weak instruments.^[26]^ Sensitivity analyses were implemented to evaluate the reliability and robustness of the causal effect of cathepsin on malignant lymphoma. The Cochran Q test was utilized to detect heterogeneity among SNPs.^[23]^ Horizontal and directional pleiotropy were recognized by the MR-PRESSO global test and MR-Egger intercept test, respectively.^[19,22]^ The leave-one-out method was employed to identify genetic IVs that may have an impact on the overall causal inference conclusions.

### 2.4. The reverse and multivariate MR analyses

In the forward MR study, targeting cathepsins that play a causal role in malignant lymphoma, a reverse MR study was initiated to investigate whether there is a reverse causal correlation between lymphoma and cathepsin. The strong and independent IVs (*P* < 5 × 10^−6^ and LD of *r*^2^ = 0.001 and kb = 10,000) that genetically related to malignant lymphoma were screened from the corresponding GWAS, with cathepsin as the outcome, and analyzed in a process similar to the forward MR study. The multivariate MR (MVMR) study is an expansion on the standard univariate MR. MVMR was performed to estimate the direct causal effect of each exposure on the outcome and is applicable in the case of estimation of the causal effect of multiple cathepsins on different subtypes of lymphoma.^[27,28]^ The conclusion of the causal Inference is established only when the results of multivariate IVW and MR-Egger methods are consistent.

### 2.5. Statistical analysis

The MR study was operated in the R environment (version 4.1.3). The R packages including “TwoSampleMR” (version 0.5.7), “MRPRESSO” (version 1.0), and “MendelianRandomization” (version 0.7.0) packages assisted in the implementation of MR analysis.

## 3. Results

### 3.1. Absence of a causal role of cathepsin in HL

**Figure 2.**
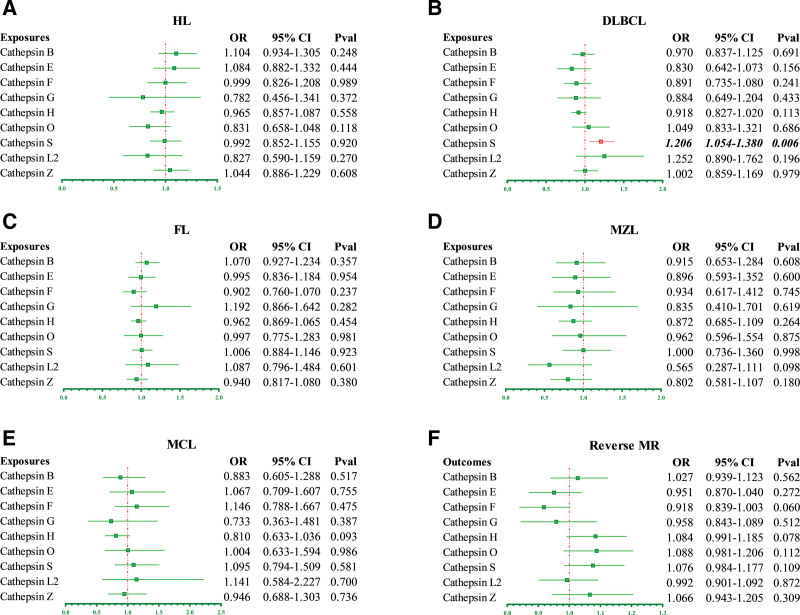
The results of a causal association between cathepsins and malignant lymphoma from MR-based analysis. (B) Genetically predicted Cathepsin S (CTSS) plays a causal role in diffuse large B cell lymphoma (DLBCL). (A, C–E) No causal association between cathepsins and other types of lymphoma. (F) Absence of reverse causality between DLBCL and CTSS. MR = Mendelian randomization

A total of 102 SNPs that strongly and independently predicted cathepsin levels (μg/L) was extracted from THE INTERVAL STUDY. One SNP (rs116142041) was eliminated as an outlier. The forest plot (Fig. 2A) demonstrated that no statistically significant causal association was found between cathepsin and HL. This means that elevated or decreased cathepsin levels are not associated with the increased risk of developing HL.

### 3.2. The causal effect of Cathepsin S on DLBCL

**Figure 3.**
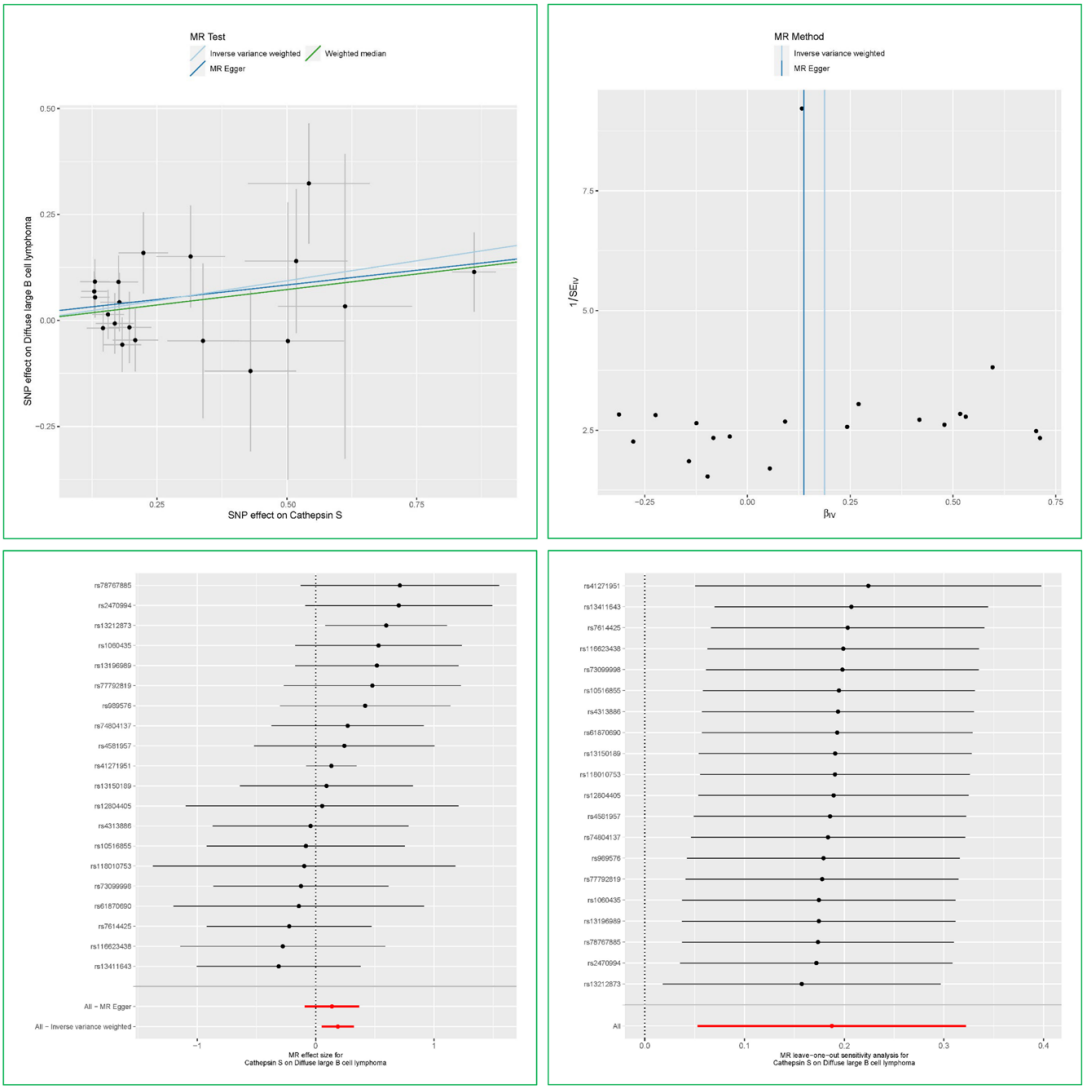
The sensitivity analysis of causal inference between Cathepsin S (CTSS) and diffuse large B cell lymphoma (DLBCL). Upper left scatter plot, upper right funnel plot, bottom left forest plot, bottom right leave-one-out method.

MR analyses were performed for each of the 4 major NHL subtypes in turn, and the results were displayed in Figure 2, and 102 genetic IVs were extracted from the cathepsin GWAS for DLBCL and 1 outlier SNP was excluded. The characteristics of the enrolled IVs were listed in Table S1, Supplemental Digital Content, https://links.lww.com/MD/Q862. As the forest plot (Fig. 2B) shows, genetically predicted Cathepsin S (CTSS) level was positively associated with the increased risk of DLBCL. Patients with elevated levels of CTSS are more susceptible to DLBCL, which corresponds to a 20.6% increased hazard (OR_IVW_ = 1.206, 95% CI 1.054–1.380, *P* = .006). Then, the MR-Robust Adjusted Profile Score method was applied to address the validity and robustness of the findings and to detect and tackle correlated pleiotropy. The results are summarized in Table 1, and this finding (OR_RAPS_ = 1.208, 95% CI 1.047–1.394, *P* = .010) supports the conclusion that there is a potential causal relationship between CTSS and DLBCL, demonstrating that the findings of this MR study are stable and reliable. The results of the sensitivity analysis of the causative relationship between CTSS and DLBCL were summarized in Table 2 and Figure 3. The scatter plot (Fig. 3, upper left) demonstrated that the risk of DLBCL showed an upward trend with the elevated CTSS level. No heterogeneity existed between the selected genetic IVs. The horizontal and directional pleiotropy were not found in the MR study by the MR-PRESSO global test and MR-Egger intercept test. The funnel plot (Fig. 3, upper right) was roughly symmetrical, demonstrating the absence of directional pleiotropy in this study. The result of the leave-one-out method was illustrated in Figure 3 (bottom right). It indicated that the arbitrary deletion of 1 SNP at a time did not affect the overall causality conclusion. Except for CTSS, no causal effect of the other 8 cathepsins on DLBCL was observed. In addition, the forward MR study also confirmed a non-causative association between cathepsin and the 3 remaining NHL subtypes.

**Table 1:** Mendelian randomization results of the MR-RAPS method.

Exposures	Outcomes	nSNPs	MR-RAPS	
			OR (95% CI)	*P*-value
Cathepsin B	DLBCL	18	0.972 (0.831–1.137)	.721
Cathepsin E	DLBCL	8	0.787 (0.624–1.004)	.054
Cathepsin F	DLBCL	9	0.884 (0.713–1.096)	.260
Cathepsin G	DLBCL	8	0.878 (0.621–1.241)	.461
Cathepsin H	DLBCL	7	0.918 (0.824–1.023)	.123
Cathepsin O	DLBCL	12	1.037 (0.808–1.331)	.776
Cathepsin S	DLBCL	20	**1.208 (1.047–1.394**)	**.010**
Cathepsin L2	DLBCL	8	1.344 (0.920–1.963)	.126
Cathepsin Z	DLBCL	12	1.032 (0.876–1.216)	.707

Bold values indicate *P*-values <.05.

CI = confidence interval, MR-RAPS = Mendelian randomization-Robust Adjusted Profile Score, nSNPs = number of single nucleotide polymorphisms, OR = odds ratio.

**Table 2:** The sensitivity analysis of the causative relationship between cathepsins and diffuse large B cell lymphoma.

Exposures/Outcomes		nSNPs	*P*-value		
			Horizontal pleiotropy	Directional pleiotropy	Heterogeneity (IVW method)
Forward MR	Cathepsin B	18	.531	.385	.473
	Cathepsin E	8	.089	.391	.048
	Cathepsin F	9	.387	.594	.353
	Cathepsin G	8	.637	.816	.585
	Cathepsin H	7	.793	.269	.897
	Cathepsin O	12	.234	.282	.235
	Cathepsin S	20	.747	.605	.710
	Cathepsin L2	8	.235	.059	.221
	Cathepsin Z	12	.263	.598	.309
Backward MR	Cathepsin B	6	.569	.610	.584
	Cathepsin E	6	.508	.983	.477
	Cathepsin F	6	.699	.372	.601
	Cathepsin G	6	.108	.987	.068
	Cathepsin H	6	.682	.989	.617
	Cathepsin O	6	.289	.294	.243
	Cathepsin S	6	.898	.606	.913
	Cathepsin L2	6	.397	.714	.330
	Cathepsin Z	6	.122	.294	.092

IVW = inverse variance weighted, MR = Mendelian randomization, nSNPs = number of single nucleotide polymorphisms.

### 3.3. No reverse causality between DLBCL and CTSS

**Figure 4.**
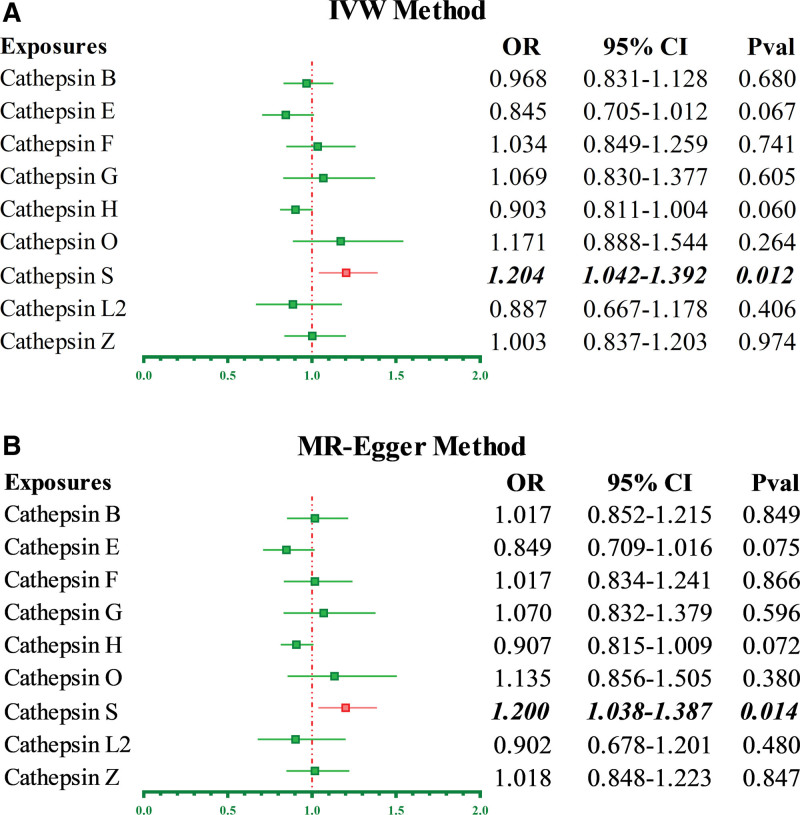
The results of multivariate Mendelian randomization (MVMR) analysis supported a positive causal link between Cathepsin S (CTSS) and diffuse large B cell lymphoma (DLBCL). (A) Multivariate inverse variance weighted method. (B) Multivariate MR-Egger method.

The characteristics of the IVs that genetically related to DLBCL were listed in Table S2, Supplemental Digital Content, https://links.lww.com/MD/Q862. Based on the backward MR analysis, it was proved that DLBCL has no causative effect on CTSS level (OR_IVW_ = 1.076, 95% CI 0.984–1.177, *P* = .109). The results of the MR analysis were recorded in the forest plot (Fig. 2F). According to Table 2, no pleiotropy and heterogeneity were detected in the reverse MR study. After MVMR analysis, the conclusion of the causal inference between DLBCL and CTSS remained valid. Both multivariate IVW and MR-Egger methods supported that elevated CTSS level is a potential hazard factor for patients with DLBCL (Fig. 4A and B, OR_IVW_ = 1.204, 95% CI 1.042–1.392, *P* = .012; OR_MR-Egger_ = 1.200, 95% CI 1.038–1.387, *P* = .014). No causal link between Cathepsin and follicular lymphoma, mantel cell lymphoma, and marginal zone lymphoma (OR_IVW_ = 1.004, 95% CI 0.998–1.211, *P* = .112; OR_IVW_ = 0.989, 95% CI 0.864–1.385, *P* = .314; OR_IVW_ = 1.215, 95% CI 0.976–1.541, *P* = .315).

## 4. Discussion

Malignant lymphomas are a heterogeneous group of hematologic malignancies that are classified into HL and NHL categories based on histopathology.^[29,30]^ At present, the etiology and pathogenesis are not fully understood. Previous studies have revealed that past infection with Epstein–Barr virus, Human immunodeficiency virus, Human T-cell lymphotropic virus type 1, and *Helicobocton Pyloni* may be associated with the pathogenesis of malignant lymphoma.^[31–34]^ With the global outbreak of severe acute respiratory syndrome coronavirus 2 in 2021, our prior study also suggested that Coronavirus disease 2019 is a potential risk factor for DLBCL.^[35]^ In addition to this, immune dysfunction plays a significant role in the development of malignant lymphoma. A wealth of evidence from observational and experimental studies indicated that immunocompromise was correlated with an increased risk of malignant lymphoma. Patients with congenital or acquired immunodeficiencies are more likely to have concomitant lymphomas than normal individuals.^[36]^ Furthermore, earlier studies have also found that patients suffering from certain autoimmune diseases on long-term immunosuppressive therapies are at increased risk of progressing to malignant lymphoma.^[37–39]^ Notably, environmental and hereditary causes also contribute to the pathogenesis of lymphomas. Recent research presents a novel perspective that long-term exposure to air pollution is connected with a higher risk of adult lymphoma. However, the above-established or potentially linked risk factors account for only a small fraction of the total incidence of lymphoma.^[40]^

Currently, R-CHOP (rituximab, cyclophosphamide, vincristine, doxorubicin, and prednisone) as the standard first-line regimen has greatly enhanced the remission rate and overall survival of most DLBCL patients and significantly improved their prognosis. However, 30% to 40% of patients still confront problems such as ineffectiveness to the R-CHOP regimen or relapse after treatment. Therefore, in recent years, novel therapeutic strategies are constantly being explored for improving the prognosis of patients with relapsed/refractory (R/R) DLBCL. These new emerging treatment options are represented by Chimeric antigen receptor T-cell therapy but also include new treatment combinations such as polatuzumab plus rituximab and bendamustine, tafasitamab plus lenalidomide, and CD3xCD20 bispecific antibodies.^[41]^ However, these new therapies are not yet very mature, and on the other hand, several severe side effects restrict their clinical application.^[42]^ Therefore, it has to be faced that it is urgent for researchers and clinicians to explore more possible etiologies and pathogenetic mechanisms of DLBCL and to develop promising new treatment strategies.

This study investigated the causal link between cathepsin and malignant lymphoma based on bidirectional and MVMR algorithms. The results suggested a positive causative relationship between genetic liability to CTSS and DLBCL. Patients with elevated CTSS levels have a 20.6% relative increased risk of developing DLBCL (OR_IVW_ = 1.206, 95% CI 1.054–1.380, *P* = .006). Besides, the causal role of the remaining cathepsins in DLBCL and other types of lymphoma was not established. No reverse causality between DLBCL and CTSS was observed after the backward MR analysis (OR_IVW_ = 1.076, 95% CI 0.984–1.177, *P* = .109). The MVMR results supported that CTSS was causally responsible for DLBCL (OR_IVW_ = 1.204, 95% CI 1.042–1.392, *P* = .012; OR_MR-Egger_ = 1.200, 95% CI 1.038–1.387, *P* = .014).

CTSS is a member of the lysosomal cysteine cathepsin family of proteases. As an endopeptidase, CTSS possesses unique properties and specific functions that distinguish it from other subfamily members. First, the expression of CTSS in tissues and cells is relatively restricted. CTSS is predominantly expressed in professional antigen-presenting cells including B lymphocytes, dendritic cells, and monocytes/macrophages. The restricted expression profile of CTTS dictates that it is primarily localized in tissues such as the spleen and the lymphatic system, where it performs the roles of antigen processing and presentation. Unlike other protease members that exert catalytic activity only in acidic lysosomal environments, CTSS is capable of self-activation via an autocatalysis process in neutral pH and even in slightly alkaline environments. This confers extracellular activity on CTSS, enabling it to participate in the hydrolysis of substrates both inside and outside the cell. CTSS expression in vivo is complexly modulated by a variety of endogenous inhibitors and regulatory proteins. Once this regulatory balance is disrupted, aberrant CTSS expression may be involved in a range of biological processes and the pathogenesis of multi-system diseases. Relevant studies have demonstrated that CTSS acts as a biological marker and potential therapeutic target for a variety of diseases under pathological conditions, including but not limited to ADs, inflammatory diseases, and malignancies.^[43]^ For instance, 1 study investigated the role of CTSS expression in breast cancer patients, and the results showed that the elevated CTSS level was positively associated with high-grade, late-stage, and poor prognostic outcomes.^[44]^ Furthermore, ASPER-29, a novel CTSS inhibitor, significantly inhibited the ability of pancreatic cancer cells to metastasize in a concentration-dependent manner. This ability to inhibit tumor cell migration and invasion was confirmed in a mouse xenograft model.^[45]^ In recent years, the emerging appearance of CTSS small molecule inhibitors and monoclonal antibodies with highly selective targeting to inhibit the proteolytic effect of CTSS provides new ways and directions for tumor treatments.^[46,47]^

The role of CTSS in DLBCL and its mechanism is less well documented. Meanwhile, the underlying pathogenic mechanism of CTSS on DLBCL remains unelucidated. Sporadic studies have noted that CTSS is overexpressed in DLBCL,^[48]^ which is consistent with the conclusions of this study. It is believed that CTSS activation is engaged in the regulation of antigen processing and T-cell-mediated immune response. Inhibition of CTSS activity impairs the interaction of tumor cells with CD4^+^ T cells in the germinal centers, leading to increased antigenic diversity and immunogenicity of lymphoma cells and promoting the recruitment and infiltration of cytotoxic T cells, which exerts a therapeutic potential in indolent and aggressive malignant lymphoma.^[48]^ These studies emphasize that the complicated modulatory network between CTSS and the tumor microenvironmentIV facilitates tumor cell invasion, migration, growth, and angiogenesis.^[49,50]^ As mentioned above, new small molecule inhibitors are constantly being developed for tumor therapies. On this basis, considering the high degree of homology among members of the lysosomal cathepsin family, more drugs with high selectivity are being explored to overcome the off-target effects of inhibitors and mitigate the side effects. Not only that, a nanocarrier-mediated drug delivery system with targeted delivery and release capabilities to promote antitumor immunity is being developed.^[51]^ In addition, inhibitory monoclonal antibodies targeting human CTSS have been designed and produced.^[47]^ Although no consensus has been achieved on the mechanisms underlying the causal effect of CTSS and DLBCL, undoubtedly, the findings of this study provide a fresh perspective on the treatment of patients with DLBCL. To date, relevant clinical trials of CTSS inhibitors are being conducted gradually. This also reminds researchers to pay more attention to the causality between CTSS and DLBCL and the potential clinical application prospect of CTSS inhibitors in DLBCL patients.

The study has certain limitations. The exposure and outcome populations had relatively small sample sizes and consisted of individuals of European ancestry, while other ethnic groups were not included in this study. Only HL and the 4 common NHL subtypes were considered for the outcome variables. Other NHL types with relatively low prevalence were not considered.

## 5. Conclusions

In conclusion, this study demonstrated a causal effect of CTSS on DLBCL based on the MR algorithm. Follow-up studies are needed to elucidate the underlying mechanisms between them and explore promising new therapeutic options for DLBCL patients.

## Acknowledgments

We want to acknowledge the participants and investigators of the FinnGen study.

## Author contributions

**Conceptualization**: Liang Yu.

**Formal analysis**: Yuye Shi, Yuan Deng.

**Methodology**: Chuanyang Lu, Qiuni Chen.

**Software**: Chuanyang Lu, Yuye Shi.

**Supervision**: Qiuni Chen.

**Validation**: Qiuni Chen, Yuan Deng, Chunling Wang.

**Visualization**: Chuanyang Lu.

**Writing – original draft**: Chuanyang Lu.

**Writing – review & editing**: Chunling Wang, Liang Yu.

## Supplementary Material
