# Supplementary material for: Cathepsins effect on diffuse large B cell lymphoma risk: A Mendelian randomization study
Source: Medicine (Baltimore). 2026 May 12;104(49):e46485. doi: 10.1097/MD.0000000000046485 (PMC12688782; doi:10.1097/MD.0000000000046485)
Supplement: Supplementary file 1 [file medi-104-e46485-s001.docx]

ST1 The characteristics of the genetic instrumental variables enrolled in the forward MR analysis.

| **Exposure** | **SNP** | **Chr** | **Position** | **Effect allele** | **Other allele** | **Beta** | **SE** | **Pval** | **Eaf** | **F statistics** |
| --- | --- | --- | --- | --- | --- | --- | --- | --- | --- | --- |
| **Cathepsin B** | rs113646963 | 2 | 204530789 | T | C | 0.200 | 0.043 | 2.95E-06 | 0.097 | 21.837 |
|  | rs11563944 | 7 | 89816334 | G | A | 0.129 | 0.028 | 3.55E-06 | 0.268 | 21.553 |
|  | rs117345475 | 11 | 131588071 | G | A | -0.433 | 0.093 | 3.47E-06 | 0.021 | 21.545 |
|  | rs117486267 | 15 | 25484738 | C | T | -0.400 | 0.083 | 1.58E-06 | 0.025 | 23.024 |
|  | rs13152767 | 4 | 122367502 | A | G | 0.416 | 0.087 | 1.62E-06 | 0.026 | 22.986 |
|  | rs143557119 | 11 | 116385842 | A | G | 0.433 | 0.095 | 4.79E-06 | 0.022 | 20.909 |
|  | rs147881440 | 17 | 80969815 | A | G | -0.233 | 0.051 | 4.79E-06 | 0.064 | 20.878 |
|  | rs148930853 | 8 | 114408661 | C | T | 0.552 | 0.118 | 3.02E-06 | 0.012 | 21.801 |
|  | rs150370599 | 11 | 60718792 | T | C | 0.218 | 0.046 | 1.78E-06 | 0.083 | 22.804 |
|  | rs150811995 | 10 | 38633385 | C | A | 0.460 | 0.100 | 4.57E-06 | 0.019 | 21.006 |
|  | rs1692819 | 8 | 11705448 | A | G | 0.425 | 0.027 | 5.25E-54 | 0.292 | 239.991 |
|  | rs200518932 | 3 | 151162527 | CA | C | -0.517 | 0.110 | 2.63E-06 | 0.017 | 22.051 |
|  | rs36021960 | 7 | 47111670 | A | G | 0.175 | 0.037 | 3.02E-06 | 0.126 | 21.806 |
|  | rs7249773 | 19 | 18289760 | A | G | 0.175 | 0.035 | 6.03E-07 | 0.151 | 24.814 |
|  | rs72863882 | 10 | 134275165 | A | G | -0.257 | 0.048 | 6.46E-08 | 0.084 | 29.233 |
|  | rs75773690 | 1 | 24464363 | A | G | -0.395 | 0.086 | 4.17E-06 | 0.022 | 21.177 |
|  | rs76089522 | 3 | 140389189 | A | G | -0.197 | 0.042 | 2.88E-06 | 0.105 | 21.905 |
|  | rs9905973 | 17 | 26735142 | A | G | -0.141 | 0.028 | 3.55E-07 | 0.271 | 25.898 |
| **CathepsinE** | rs10900907 | 6 | 1448387 | A | G | 0.119 | 0.026 | 3.24E-06 | 0.417 | 21.691 |
|  | rs112918835 | 8 | 109846123 | T | C | 0.706 | 0.153 | 3.72E-06 | 0.007 | 21.403 |
|  | rs13089837 | 3 | 5094952 | C | T | -0.115 | 0.025 | 4.17E-06 | 0.498 | 21.221 |
|  | rs189009983 | 4 | 102582604 | T | C | 0.706 | 0.154 | 4.68E-06 | 0.008 | 20.947 |
|  | rs57135345 | 10 | 26671589 | T | C | 0.254 | 0.054 | 2.95E-06 | 0.057 | 21.862 |
|  | rs57689619 | 4 | 189431921 | G | A | -0.208 | 0.044 | 2.82E-06 | 0.086 | 21.891 |
|  | rs74677283 | 11 | 19486084 | T | C | 0.438 | 0.088 | 6.76E-07 | 0.022 | 24.635 |
|  | rs8066936 | 17 | 754600 | A | G | -0.149 | 0.033 | 4.27E-06 | 0.179 | 21.062 |
| **Cathepsin F** | rs10745925 | 12 | 102218899 | C | T | -0.287 | 0.027 | 9.12E-27 | 0.294 | 114.932 |
|  | rs112526544 | 7 | 26300072 | G | A | 0.380 | 0.077 | 6.92E-07 | 0.028 | 24.608 |
|  | rs115901379 | 2 | 106335628 | G | T | 0.431 | 0.093 | 3.39E-06 | 0.020 | 21.564 |
|  | rs1260326 | 2 | 27730940 | C | T | -0.164 | 0.025 | 6.92E-11 | 0.598 | 42.431 |
|  | rs143015877 | 13 | 23327148 | T | C | -0.133 | 0.029 | 3.98E-06 | 0.257 | 21.301 |
|  | rs183683891 | 6 | 133102456 | T | C | -0.524 | 0.112 | 3.02E-06 | 0.015 | 21.776 |
|  | rs186369051 | 5 | 93830000 | T | C | -0.482 | 0.103 | 2.63E-06 | 0.015 | 22.038 |
|  | rs61866943 | 10 | 85428041 | G | T | 0.497 | 0.101 | 9.12E-07 | 0.019 | 24.094 |
|  | rs7564167 | 2 | 645190 | G | A | -0.265 | 0.053 | 4.90E-07 | 0.936 | 25.347 |
| **Cathepsin G** | rs114418234 | 2 | 155060275 | C | A | 0.308 | 0.065 | 2.24E-06 | 0.037 | 22.396 |
|  | rs116142041 | 4 | 9787836 | A | G | -0.336 | 0.067 | 4.57E-07 | 0.036 | 25.453 |
|  | rs117133380 | 14 | 107206586 | A | C | 0.500 | 0.107 | 2.63E-06 | 0.016 | 22.046 |
|  | rs147099093 | 3 | 37730576 | A | G | 0.427 | 0.089 | 1.38E-06 | 0.021 | 23.276 |
|  | rs35241999 | 12 | 11184504 | G | A | 0.406 | 0.087 | 2.95E-06 | 0.024 | 21.804 |
|  | rs4702448 | 5 | 7295725 | A | C | 0.128 | 0.027 | 1.45E-06 | 0.666 | 23.135 |
|  | rs497459 | 7 | 49838522 | C | T | -0.175 | 0.037 | 1.95E-06 | 0.865 | 22.652 |
|  | rs72773561 | 10 | 9677794 | C | T | -0.253 | 0.053 | 1.70E-06 | 0.057 | 22.928 |
| **Cathepsin H** | rs12911554 | 15 | 51542757 | T | C | -0.120 | 0.025 | 1.82E-06 | 0.550 | 22.767 |
|  | rs146037740 | 18 | 11507434 | A | G | 0.474 | 0.093 | 3.47E-07 | 0.023 | 25.973 |
|  | rs147991203 | 4 | 140256765 | T | C | 0.376 | 0.077 | 1.17E-06 | 0.027 | 23.621 |
|  | rs34843303 | 15 | 79234470 | C | T | -1.119 | 0.034 | ######## | 0.114 | 1058.255 |
|  | rs35628511 | 1 | 24667702 | T | C | -0.125 | 0.027 | 4.17E-06 | 0.312 | 21.208 |
|  | rs60018174 | 16 | 5452751 | T | C | 0.196 | 0.040 | 7.24E-07 | 0.116 | 24.506 |
|  | rs62013235 | 15 | 79246166 | A | G | 0.402 | 0.043 | 1.35E-20 | 0.091 | 86.541 |
| **Cathepsin O** | rs10902420 | 12 | 132150183 | G | A | -0.210 | 0.043 | 1.23E-06 | 0.899 | 23.549 |
|  | rs146963690 | 2 | 156493388 | G | T | 0.625 | 0.132 | 2.29E-06 | 0.010 | 22.309 |
|  | rs149159018 | 3 | 132077351 | G | A | 0.703 | 0.134 | 1.55E-07 | 0.011 | 27.538 |
|  | rs17288007 | 4 | 159878046 | G | A | -0.246 | 0.052 | 2.45E-06 | 0.064 | 22.208 |
|  | rs181844705 | 5 | 75333560 | G | A | 0.441 | 0.091 | 1.10E-06 | 0.023 | 23.753 |
|  | rs2439803 | 11 | 129083967 | G | A | 0.176 | 0.035 | 5.13E-07 | 0.153 | 25.213 |
|  | rs4076941 | 11 | 11261567 | C | T | -0.125 | 0.026 | 2.14E-06 | 0.369 | 22.567 |
|  | rs4297371 | 10 | 11249367 | C | A | 0.145 | 0.031 | 3.55E-06 | 0.793 | 21.537 |
|  | rs4843804 | 16 | 88156187 | A | G | -0.312 | 0.067 | 3.47E-06 | 0.034 | 21.534 |
|  | rs7140599 | 14 | 39942467 | C | T | -0.122 | 0.025 | 1.17E-06 | 0.577 | 23.572 |
|  | rs78943701 | 10 | 73736674 | A | G | -0.479 | 0.103 | 2.95E-06 | 0.015 | 21.825 |
|  | rs9932172 | 16 | 7642591 | T | C | 0.192 | 0.040 | 1.74E-06 | 0.108 | 22.792 |
| **Cathepsin S** | rs10516855 | 4 | 90924478 | C | T | -0.197 | 0.042 | 2.95E-06 | 0.096 | 21.861 |
|  | rs1060435 | 11 | 68855595 | G | A | -0.129 | 0.025 | 2.75E-07 | 0.398 | 26.439 |
|  | rs116623438 | 5 | 102689283 | C | T | -0.430 | 0.088 | 9.12E-07 | 0.020 | 24.080 |
|  | rs118010753 | 21 | 18833920 | C | T | 0.502 | 0.108 | 3.47E-06 | 0.014 | 21.535 |
|  | rs12804405 | 11 | 24422062 | A | G | 0.612 | 0.129 | 2.09E-06 | 0.010 | 22.494 |
|  | rs13150189 | 4 | 181239467 | A | G | 0.156 | 0.031 | 3.47E-07 | 0.211 | 25.841 |
|  | rs13196989 | 6 | 239373 | T | C | -0.176 | 0.037 | 2.57E-06 | 0.131 | 22.082 |
|  | rs13212873 | 6 | 143059208 | C | T | 0.542 | 0.117 | 3.55E-06 | 0.012 | 21.463 |
|  | rs13411643 | 2 | 60351132 | C | T | 0.183 | 0.036 | 5.01E-07 | 0.137 | 25.288 |
|  | rs2470994 | 7 | 47430626 | C | T | 0.130 | 0.028 | 3.39E-06 | 0.281 | 21.522 |
|  | rs41271951 | 1 | 150737220 | G | A | -0.861 | 0.042 | 7.08E-94 | 0.083 | 421.513 |
|  | rs4313886 | 18 | 74370742 | C | T | 0.169 | 0.037 | 3.89E-06 | 0.134 | 21.349 |
|  | rs4581957 | 2 | 174866439 | A | G | -0.178 | 0.037 | 1.15E-06 | 0.134 | 23.635 |
|  | rs61870690 | 10 | 127523966 | G | A | -0.339 | 0.068 | 7.24E-07 | 0.041 | 24.491 |
|  | rs73099998 | 12 | 58633987 | T | C | -0.146 | 0.031 | 2.19E-06 | 0.208 | 22.456 |
|  | rs74804137 | 12 | 18017954 | C | T | -0.518 | 0.099 | 1.58E-07 | 0.018 | 27.506 |
|  | rs7614425 | 3 | 98174258 | A | G | -0.208 | 0.044 | 1.82E-06 | 0.093 | 22.789 |
|  | rs77792819 | 12 | 94386245 | G | A | 0.315 | 0.066 | 1.95E-06 | 0.041 | 22.653 |
|  | rs78767885 | 19 | 18112020 | C | T | 0.224 | 0.048 | 2.69E-06 | 0.087 | 22.000 |
|  | rs989576 | 4 | 127182592 | T | C | 0.131 | 0.028 | 2.57E-06 | 0.298 | 22.090 |
| **Cathepsin L2** | rs116407656 | 4 | 68099087 | C | T | 0.401 | 0.086 | 2.75E-06 | 0.024 | 21.976 |
|  | rs117714361 | 12 | 109142132 | G | A | -0.345 | 0.076 | 4.90E-06 | 0.027 | 20.837 |
|  | rs13068566 | 3 | 102862667 | G | A | 0.116 | 0.025 | 2.88E-06 | 0.466 | 21.929 |
|  | rs146867532 | 10 | 43225400 | A | AT | -0.336 | 0.071 | 2.09E-06 | 0.967 | 22.455 |
|  | rs151179824 | 3 | 173226009 | A | G | 0.445 | 0.094 | 2.34E-06 | 0.022 | 22.245 |
|  | rs2302837 | 17 | 6014176 | G | A | -0.254 | 0.050 | 3.31E-07 | 0.925 | 26.019 |
|  | rs34635662 | 7 | 27220271 | CT | C | 0.229 | 0.050 | 3.89E-06 | 0.077 | 21.315 |
|  | rs7898416 | 10 | 83700161 | G | A | -0.228 | 0.049 | 3.24E-06 | 0.071 | 21.669 |
| **Cathepsin Z** | rs10745925 | 12 | 102218899 | C | T | -0.362 | 0.027 | 2.29E-42 | 0.294 | 185.503 |
|  | rs10761760 | 10 | 65179152 | C | A | 0.132 | 0.026 | 3.09E-07 | 0.449 | 26.200 |
|  | rs1135945 | 4 | 516586 | A | G | 0.139 | 0.030 | 4.17E-06 | 0.208 | 21.159 |
|  | rs114675081 | 3 | 106826623 | G | A | 0.515 | 0.108 | 1.66E-06 | 0.015 | 22.964 |
|  | rs116920068 | 10 | 54555095 | A | G | -0.350 | 0.075 | 3.02E-06 | 0.029 | 21.798 |
|  | rs148370779 | 20 | 57602212 | T | C | -1.763 | 0.111 | 2.57E-57 | 0.013 | 254.458 |
|  | rs298724 | 9 | 94023321 | C | T | 0.248 | 0.054 | 3.98E-06 | 0.055 | 21.270 |
|  | rs36128387 | 1 | 235460258 | T | C | 0.439 | 0.091 | 1.55E-06 | 0.023 | 23.055 |
|  | rs4761709 | 12 | 93785170 | G | A | 0.122 | 0.025 | 1.00E-06 | 0.409 | 23.913 |
|  | rs67845377 | 19 | 19275802 | T | C | 0.168 | 0.036 | 3.72E-06 | 0.139 | 21.397 |
|  | rs7656806 | 4 | 138271322 | C | A | -0.128 | 0.025 | 1.86E-07 | 0.516 | 27.109 |
|  | rs770140 | 5 | 19430825 | G | A | -0.124 | 0.027 | 2.82E-06 | 0.642 | 21.917 |

ST2 The characteristics of the genetic instrumental variables enrolled in the reverse MR analysis.

| **Exposure** | **SNP** | **Position** | **Effect allele** | **Other allele** | **Beta** | **SE** | **Pval** | **Eaf** | **F Statistics** |
| --- | --- | --- | --- | --- | --- | --- | --- | --- | --- |
| **DLBCL** | rs10879771 | 74423549 | A | C | 0.232 | 0.049 | 2.15E-06 | 0.251 | 22.457 |
|  | rs13255292 | 128064327 | T | C | 0.234 | 0.046 | 2.65E-07 | 0.342 | 26.49 |
|  | rs2269720 | 57724465 | C | T | -0.224 | 0.048 | 2.62E-06 | 0.351 | 22.075 |
|  | rs4713570 | 32658263 | T | C | 0.36 | 0.046 | 4.74E-15 | 0.294 | 61.366 |
|  | rs67946412 | 15322346 | C | A | -0.225 | 0.048 | 2.20E-06 | 0.348 | 22.413 |
|  | rs80052472 | 32865956 | A | C | 0.396 | 0.086 | 3.69E-06 | 0.059 | 21.417 |
